# Outcomes of Community Input on Research Recruitment at an Alzheimer’s Disease Research Center

**DOI:** 10.1002/alz70861_108914

**Published:** 2025-12-23

**Authors:** Antoine R Trammell

**Affiliations:** ^1^ Emory University Goizueta Alzheimer's Disease Research Center, Atlanta, GA USA

## Abstract

**Background:**

Per prevalence estimates, Alzheimer’s disease and related dementias (ADRD) will affect over 14 million older US adults by 2060. ADRD risk is unequal, as older Black/African American (AA) and Hispanic American (HA) adults have a higher risk than their non‐Hispanic White (NHW) counterparts. Given this difference, future disease burden estimates are less precise as data from cohorts reflective of the general population are lacking. Improving research sample heterogeneity is crucial for more accurate data to estimate ADRD population trends.

**Method:**

We examined participation histories for all newly enrolled participants in the Emory Goizueta Alzheimer’s Disease Research Center (GADRC) Uniform Data Set (UDS) cohort between January 1, 2016, and January 16, 2024. We compared AA and NHW by demographics and pre‐consent activities using t‐tests and chi‐square. Next, we examined associations between demographics and activity participation before UDS consent with Generalized Estimating Equations.

**Result:**

We analyzed data for 194 people (74.7 (9.1) years, 16 (14‐18) education years, and 59% female). AA averaged more activities than NHW, 2(1‐4) vs. 0 (0.2), *p* <.01, and 41% of AA men attended dedicated men’s health programming. Compared to NHW, AA enrolled in research faster (363 vs.1097 days, *p* <.01). AA were more likely to attend in‐person events before consent (OR: 7.02, 95% CI: 3.57,18.84), and first contact to enrollment was 640 days faster (95% CI: ‐985, ‐295) than NHW. AA males enrolled in research 508 days faster (95% CI: ‐682, ‐334) than AA females after first contact.

**Conclusion:**

Our center’s recruitment approach utilizes community input to tailor in‐person programming content, establish tangible outcomes, and facilitate direct interaction with researchers. We also developed intentional programming for specific demographics that led to increased AA male enrollment. These results offer tangible lessons on approaching research recruitment to enhance study sample heterogeneity.

